# Coffee intake and the vasopressin system: an epidemiological and experimental study

**DOI:** 10.1530/EC-25-0100

**Published:** 2025-09-05

**Authors:** Fredrika Schill, Simon Timpka, Sophie Hellstrand, Olle Melander, Sofia Enhörning

**Affiliations:** ^1^ Department of Cardiology, Skåne University Hospital, Malmö, Sweden; ^2^ Perinatal and Cardiovascular Epidemiology, Department of Clinical Sciences Malmö, Lund University, Malmö, Sweden; ^3^ Department of Obstetrics and Gynecology, Skåne University Hospital, Malmö, Sweden; ^4^ Department of Clinical Sciences, Lund University, Malmö, Sweden; ^5^ Department of Internal Medicine, Skåne University Hospital, Malmö, Sweden

**Keywords:** copeptin, vasopressin, coffee, hydration

## Abstract

Coffee is epidemiologically linked to health benefits and risks. Coffee is thought to be a diuretic. However, it can still contribute to daily fluid intake. Vasopressin is the most important physiological regulator of body fluid balance and diuresis. This study aimed to map the effects of coffee intake on vasopressin concentration. In the population-based cross-sectional Malmö Offspring Study (*n* = 3,270, age 18–75 years, 47% males) we performed linear regression analyses to investigate the association between coffee intake and plasma concentration of copeptin (a vasopressin surrogate marker). Coffee intake was assessed using a 4-day food record. Moreover, we compared plasma copeptin concentrations after intake of 4 dL of coffee and 10 mL of water (control) in an experimental study (*n* = 26, age 35–70 years, 15% males). Results showed that higher coffee intake was associated with lower copeptin concentration after adjusting for co-variables, including total fluid intake. In the coffee experiment, the acute intake of 4 dL of coffee significantly decreased copeptin concentration at all time points (every 30 min for 4 h) compared with baseline concentration. A 27% maximum reduction on average was observed within 150 min. Intake of 10 mL of water also resulted in a slight reduction of copeptin concentration within 2 h. These findings suggest that copeptin concentration is lower among individuals with high coffee intake and can be acutely decreased by coffee intake. The mechanisms behind the coffee-induced reduction in copeptin concentration may involve oral and gut reflexes, volume load, and/or specific effects of coffee compounds.

## Introduction

Coffee is one of the most widely consumed beverages worldwide. Aside from water, a cup of coffee contains a large number of biologically active compounds, including caffeine, polyphenols, melanoidins, and minerals (e.g., potassium, calcium, and magnesium) (1). Epidemiological studies have comprehensively investigated the physiological effects of coffee and its impact on various diseases. Coffee intake has been linked to cardiovascular, metabolic, neurological, and musculoskeletal diseases, and pregnancy outcomes. Although the findings are somewhat conflicting, moderate coffee intake (2–5 cups a day) seems to be generally safe in healthy adults, and there are indications of beneficial associations with several diseases (2, 3, 4, 5, 6, 7, 8, 9, 10).

Caffeine is the most studied compound in coffee. Caffeine is thought to be responsible for the increased mental alertness experienced by many coffee drinkers (11). Moreover, it has been found to increase diuresis, an effect that is most often explained by the antagonizing effects on renal adenosine receptors (12). However, the precise renal effects of caffeine are not fully understood (13, 14).

Regardless of the effects of its smaller compounds, water is the major ingredient in a cup of coffee. Although the possible physiological effects of coffee have mainly been attributed to other ingredients, the possible effect of the extra water intake that comes with coffee intake has been less studied. Depending on local customs, every cup of coffee adds approximately 0.5–4 dL to the total fluid intake. In Sweden, filtered or boiled coffee with high water content is commonly consumed (15). However, since coffee has been proposed to have diuretic properties, the net effect on hydration status is debated (16, 17).

The hydration status, or rather plasma osmolality, is tightly regulated by the vasopressin system. Vasopressin stimulates water reuptake in the kidneys through vasopressin 2 receptors (V2R), which decrease plasma osmolality. During high plasma osmolality and low body water content, vasopressin is released; in these conditions, vasopressin can easily be reduced by drinking water (18).

Vasopressin concentration is often measured by analyzing copeptin (a vasopressin precursor peptide). Copeptin has been shown to correspond well with vasopressin levels and can be reliably measured in plasma (19). Although the diuretic effect of coffee is most often attributed to adenosine receptor inhibition, some studies suggest an interplay between caffeine and the vasopressin system on diuresis (13). However, no study has directly investigated the link between coffee intake and vasopressin concentration.

The aim of this study was to investigate the possible effects of coffee intake on vasopressin, measured as plasma copeptin concentration, in an observational population-based setting and during a short-term coffee experiment.

## Methods

**Figure 1:**
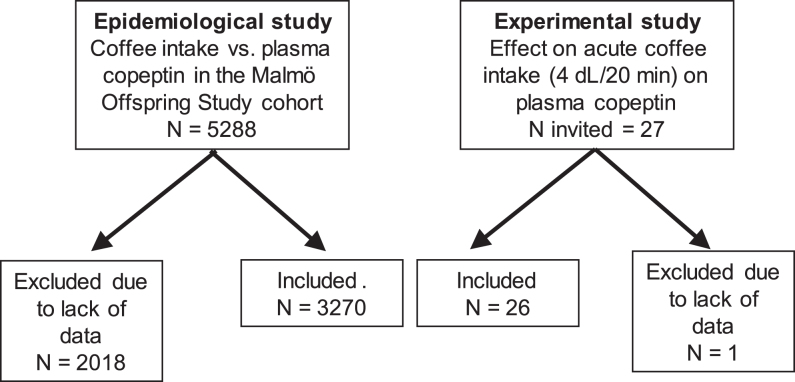
Overview of study designs.

This study has two methodologically different parts (one epidemiological and one experimental), with the overall goal to map the effects of coffee intake on copeptin concentration. The study designs are illustrated in Fig. 1.

### Method of the observational study: the Malmö Offspring Study (MOS)

#### Study population

We used data from the MOS, a population-based cohort with baseline examinations performed between 2013 and 2021 (20). In this study, 5,288 adults (age >18 years), children and grandchildren of participants in the Malmö Diet and Cancer – Cardiovascular Cohort were recruited (16). Anthropometric measurements were taken with the participants wearing indoor clothing without shoes and hats. The height (centimeters) was examined with participants standing with their legs together and looking straight ahead. Weight (kilograms) was measured on a calibrated balance beam or digital scale. Brachial systolic and diastolic blood pressure (millimeters of mercury) and heart rate (beats/minute) were measured as a mean of two measurements in the supine position after 10 min of rest by use of an automatic device (Omron, Japan). Fasting blood samples were collected and analyzed for lipids (triglycerides, high-density lipoprotein (HDL), and low-density lipoprotein (LDL)), fasting glucose, and creatinine at the Department of Clinical Chemistry, Malmö. Additional plasma samples were frozen and stored at −80°C in a local biobank for later analysis of copeptin, which was analyzed using a KRYPTOR Compact PLUS device and a commercially available chemiluminescence sandwich immunoassay Copeptin proAVP kit with coated tubes (Thermo Scientific B·R·A·H·M·S Copeptin proAVP KRYPTOR, USA). Lifestyle parameters were assessed using an extensive web-based questionnaire (20). Dietary intake was assessed through a validated web-based 4-day food record (‘Riksmaten 2010’) designed by the Swedish National Food Agency (21), wherein all consumed foods and beverages were recorded for 4 consecutive days. The participants were instructed to start recording the same day as they were included and provided with a notebook and photo book with portion sizes to make the registration as easy and correct as possible. The registered dietary intake was converted into total energy, nutrients, and water by using the National Food Database (Riksmaten vuxna 2010, version 10-05-05; https://researchdata.se/en/catalogue/dataset/ext0093-1). The total coffee intake included all types of coffee (filtered, instant, boiled, and espresso) and was based on self-registered cups converted into mean intake in grams per day. Drinking water intake included self-registered intake of tap and mineral water and was calculated as mean intake in grams per day. The total fluid intake included water in beverages, including coffee, and food moisture and was calculated as mean intake in grams per day.

Before signing informed consent, all individuals were given written and oral information about the study. Ethical approval was obtained from the regional ethics committee in Lund (registration number 2012/594). The study was performed in line with the ethical standards laid down in the 1964 Declaration of Helsinki and its later amendments and other relevant guidelines.

#### Statistics

Baseline characteristics were analyzed. They were described as absolute counts and proportions for categorical variables, and means or medians (standard deviation (SD) or 25th–75th percentile) for continuous variables, depending on the distribution of data. Coffee consumption was divided into tertiles because of an expected number of zero drinkers and a non-normally distributed consumption among coffee drinkers. Sex-specific tertiles of coffee intake, drinking water intake, and total fluid intake were used throughout, since water intake, dietary patterns, and self-reported dietary intake tend to differ according to sex (22, 23, 24). Since copeptin is known to be non-normally distributed, the natural logarithm (ln) of copeptin was used for all regression analyses.

The association between coffee intake and copeptin was tested in a linear regression analysis with copeptin as the dependent variable. The model was adjusted for age and sex (model 2). Additional adjustments were made for creatinine, HDL, LDL, triglycerides, glucose, systolic blood pressure, body mass index (BMI), and smoking (model 3); drinking water intake (model 4); and total fluid intake (model 5). The association between coffee intake and copeptin was also analyzed in strata (tertiles) of drinking water intake and total fluid intake. Data from all linear regression analyses were expressed as beta (95% confidence intervals), unit increase in the dependent variable (copeptin) per tertile increase in coffee intake. The significance level was set to be covered by a 95% confidence interval.

### Method of the experimental study

#### Study population and procedure description

Thirty-nine subjects were recruited between 2011 and 2016. The participants were found through advertisements in local press, advertisements directed toward staff at Lund University, and telephone contacts with the participants of two previous population-based cohort studies performed in Malmö (25, 26). The study was conducted in accordance with the guidelines laid down in the Declaration of Helsinki. All procedures involving human subjects were approved by the regional ethics committee in Lund (registration number 2010/740). Written informed consent was obtained from all subjects. The trial was registered at ClinicalTrials.gov (‘Water and Coffee Intervention in Humans’, NCT06165185; https://clinicaltrials.gov/study/NCT06165185?term=NCT06165185).

Each participant underwent three acute intervention procedures: 1 L of water, 4 dL of coffee, or 10 mL of water (control). The results from the water intervention have already been described and published previously (18). Each participant underwent the procedures in random order, and each procedure was separated by a washout period of 3 weeks. In this study, we analyzed the coffee and control procedures. The coffee ingested during the coffee experiment comprised commercial instant coffee powder (Nestlé Nescafé instant coffee). For each deciliter of coffee, 7.5 mL (∼1.75 g) of coffee powder was mixed with 1 dL of tap water.

Upon arriving at the clinical research unit, fasting sampling for urine osmolality, plasma osmolality, plasma sodium, plasma potassium, and plasma copeptin was performed. Thereafter, the participants ingested 4 dL of coffee (coffee procedure) or 10 mL of water (control procedure) for a maximum of 20 min. Afterward, repeated blood sampling for plasma copeptin analysis was performed every 30 min for 4 h.

#### Statistics

All laboratory measurements are described as absolute counts and proportions for categorical variables, and means (SD) or medians (25th–75th percentile) for continuous variables, depending on the distribution of data. The experimental data are presented as median (25th–75th percentile) plasma copeptin concentration at different times (0, 30, 60, 90, 120, 150, 180, 210, and 240 min) from the coffee or control procedure.

The median plasma copeptin concentration at different time points was compared with the baseline value (time 0) for both groups. The change from baseline was tested using Wilcoxon signed-rank test. Furthermore, we performed the analysis in groups of higher (≥400 mOsm/kg H_2_O) and lower urine osmolality (<400 mOsm/kg H_2_O) to investigate copeptin response to coffee at various levels of hydration. The significance level was set to be covered by a 95% confidence interval.

## Results

### Results from the MOS

Complete data were available in 3,270 participants and used for further analysis. Participants’ baseline characteristics by sex-specific tertile of coffee intake are shown in Table 1. The participants in the highest tertile of coffee intake had a coffee intake of 575 mL (in median) per day. Moreover, they tended to be older, male smokers, and to have a higher BMI, LDL, and blood pressure (systolic and diastolic) compared with those in the lowest tertile. The participants in the highest tertile of coffee intake also had a greater degree of drinking water intake and total fluid intake, and lower copeptin concentration compared with those in the lowest tertile. The individuals in the lowest tertile constituted mostly zero-coffee consumers.

**Table 1:** Baseline characteristics (*n* = 3,270).

	Coffee tertile 1	Coffee tertile 2	Coffee tertile 3
	(*n* = 1,081)	(*n* = 1,098)	(*n* = 1,091)
Coffee intake, g/day*	0.0 (0.0, 18.8)	275.0 (212.5, 350.0)	575.0 (475.0, 700.0)
Coffee intake men, g/day*	0.0 (0.0, 50.0)	300.0 (225.0, 375.0)	600 (510.0, 775.0)
Coffee intake women, g/day*	0.0 (0.0, 0.0)	275.0 (200.0, 337.0)	537.0 (450.0, 650.0)
Copeptin, pmol/L*	6.4 (4.2, 10.1)	5.7 (3.8, 8.9)	5.3 (3.6, 8.0)
Copeptin men, pmol/L*	8.1 (5.7, 12.5)	7.3 (5.0, 11.0)	6.7 (3.4, 9.4)
Copeptin women, pmol/L*	5.1 (3.4, 8.0)	4.6 (3.3, 6.6)	4.2 (3.2, 6.3)
Drinking water intake, g/day*	525 (169, 1,000.00)	550 (248, 925)	675 (325, 1,075)
Total fluid intake, g/day	1,989 (851)	2,118 (746)	2,574 (746)
Age, years	36.0 (14.2)	45.6 (14.2)	47.9 (12.5)
Men, *n* (%)			
Current smoking, *n* (%)^†^	109 (10.7)	129 (12.2)	167 (15.9)
Glucose, mmol/L	5.3 (0.8)	5.4 (1.2)	5.4 (1.1)
Creatinine, μmol/L	77.3 (14.5)	78.3 (22.3)	77.3 (13.9)
BMI, kg/m^2^	25.5 (4.9)	26.1 (4.6)	26.4 (4.4)
HDL, mmol/L	1.6 (0.4)	1.7 (0.5)	1.7 (0.5)
LDL, mmol/L	3.0 (0.9)	3.2 (1.0)	3.3 (1.0)
Triglycerides, mmol/L	1.1 (0.6)	1.2 (0.8)	1.1 (0.6)
Systolic blood pressure, mmHg	115.1 (15.3)	120.9 (16.4)	121.7 (15.1)
Diastolic blood pressure, mmHg	71.5 (9.7)	75.3 (9.9)	76.2 (9.7)

Sex-specific tertiles of coffee intake were used.

Data expressed as mean (standard deviation) if nothing else specified.

* Median (25th, 75th percentile).

^†^ *n* = 3,133.

A comparison of the baseline characteristics between included and excluded individuals is shown in Supplemental Table 1 (see section on Supplementary materials given at the end of the article). The excluded participants were comparable to the included participants in terms of age, sex, and metabolic factors. Moreover, the excluded participants had slightly higher median copeptin concentration, slightly lower median coffee intake and drinking water intake, and higher frequency of smokers compared with the included participants.

We found a significant association between increasing sex-specific coffee intake tertile and decreasing copeptin concentration (*P* < 0.001) (Table 2). In addition, the significance was maintained after adjusting for relevant confounders (e.g., age, sex, creatinine, HDL, LDL, triglycerides, glucose, systolic blood pressure, BMI, and smoking) and either drinking water intake or fluid intake. When the analysis was stratified on drinking water intake and total fluid intake (sex-specific tertiles, respectively), a significant association between coffee intake and copeptin was found only in the two lowest tertiles of drinking water intake and the lowest tertile of total fluid intake (i.e., among individuals with the lowest habitual fluid intake) after adjusting for age and sex (Table 3).

**Table 2:** Association between coffee intake and copeptin concentration in the Malmö Offspring Study (*n* = 3,270).

	Per coffee tertile increase (beta (CI))*	*P*	Coffee tertile 2 vs 1 (beta (CI))^†^	*P*	Coffee tertile 3 vs 1 (beta (CI))^‡^	*P*
Model 1	−0.10 (−0.13–−0.07)	<0.001	−0.10 (−0.16–−0.04)	<0.001	−0.19 (−0.25–−0.14)	<0.001
Model 2	−0.09 (−0.11–−0.06)	<0.001	−0.09 (−0.14–−0.035)	0.001	−0.17 (−0.23–−0.12)	<0.001
Model 3	−0.08 (−0.11–−0.05)	<0.001	−0.09 (−0.14–0.03)	0.003	−0.16 (−0.22–−0.11)	<0.001
Model 4	−0.07 (−0.10–−0.04)	<0.001	−0.08 (−0.14–−0.03)	0.004	−0.14 (−0.20–−0.08)	<0.001
Model 5	−0.03 (−0.06–−0.001)	0.045	−0.06 (−0.11–−0.0053)	0.03	−0.06 (–0.12–−0.0102)	0.042

* Data expressed as unit change in ln-transformed copeptin per tertile increase in coffee intake.

^†^ Data expressed as unit change in ln-transformed copeptin if belonging to coffee tertile 2 instead of coffee tertile 1.

^‡^ Data expressed as unit change in ln-transformed copeptin if belonging to coffee tertile 3 instead of coffee tertile 1.

Models:

Model 1: crude.

Model 2: adjusted for age and gender.

Model 3: adjusted for age, sex, creatinine, HDL, LDL, triglycerides, glucose, systolic blood pressure, BMI, and smoking.

Model 4: adjusted for age, sex, creatinine, HDL, LDL, triglycerides, glucose, systolic blood pressure, BMI, smoking, and drinking water intake.

Model 5: adjusted for age, sex, creatinine, HDL, LDL, triglycerides, glucose, systolic blood pressure, BMI, smoking, and total fluid intake.

*n* = 3,133 for models 3–5.

**Table 3:** Association between coffee intake and copeptin concentration in strata of drinking water intake and total fluid intake (*n* = 3,270).

	Per coffee tertile increase (beta (CI))	*P*	Coffee tertile 2 vs 1 (beta (CI))	*P*	Coffee tertile 3 vs 1 (beta (CI))	*P*
**Strata of drinking water intake (sex specific tertiles)**						
Tertile 1	−0.13 (−0.17–−0.08)	<0.001	−0.11 (−0.20–−0.02)	0.01	−0.25 (−0.35–−0.15)	<0.001
Tertile 2	−0.07 (−0.12–−0.02)	0.003	−0.11 (−0.20–−0.18)	0.02	−0.14 (−0.24–−0.05)	0.003
Tertile 3	−0.03 (−0.08–0.02)	0.25	−0.03 (−0.13–0.07)	0.60	−0.06 (−0.16–−0.04)	0.25
**Strata of total fluid intake (sex specific tertiles)**						
Tertile 1	−0.07 (−0.13–−0.02)	0.01	−0.09 (−0.18–−0.01)	0.03	−0.13 (−0.25–−0.02	0.02
Tertile 2	−0.04 (−0.09–0.01)	0.10	−0.07 (−0.16–0.03)	0.18	−0.08 (−0.18–−0.01)	0.09
Tertile 3	−0.01 (−0.06–0.04)	0.77	−0.02 (−0.13–0.09)	0.74	−0.02 (−0.11–0.08)	0.74

Data expressed as unit change in ln-transformed copeptin per tertile increase in coffee intake.

Median (25th, 75th percentile) drinking water intake (mL) was 75 (0.175) for men and 200 (25, 325) for women in tertile 1; 488 (375, 600) for men and 662 (550, 775) for women in tertile 2; 1,200 (925, 1,594) for men and 1,250 (1,050, 1,550) for women in tertile 3.

Mean (SD) total fluid intake (mL) was 1,446 (344) for men and 1,391 (306) for women in tertile 1; 2,229 (190) for men and 2,084 (173) for women in tertile 2; 3,266 (729) for men and 2,980 (554) for women in tertile 3.

All analyses were adjusted for age and gender.

### Results from the experimental study

Out of the 39 initially intended participants, only 27 were invited and completed the coffee procedure. This is because the recruitment to the coffee experiment was stopped after including 27 participants due to frequent gastrointestinal side effects. Of the 27 participants with data from the coffee and control procedures, one participant was excluded from analysis because of lack of a first copeptin measurement before the start of the coffee procedure. Most participants were women (85%), and the mean age was 60 years. The median copeptin concentration was slightly higher at the start of the coffee procedure compared with that of the control procedure (4.88 and 3.76 pmol/L, respectively). The baseline values of urine and plasma samples in both procedures are shown in Table 4.

**Table 4:** Mean/median concentration of urine (u-) and plasma (p-) samples at baseline of coffee intervention and control procedure (*n* = 26).

	Start of coffee intervention	Start of control procedure
P-copeptin (pmol/L)*	4.88 (3.40–7.26)	3.76 (3.06–6.31)
U-osmolality (mOsm/kg H_2_O)*	384.0 (315.8–567.3)	400.5 (314.3–551.5)
P-osmolality (mOsm/kg)^†^	292.96 (4.46)	293.00 (3.96)
P-sodium (mmol/L)^†^	140.96 (1.68)	140.54 (1.58)
P-potassium (mmol/L)^†^	3.90 (0.22)	3.88 (0.22)
P-creatinine (pmol/L)^†^	72.39 (12.45)	73.23 (11.62)

* Data are expressed as median (25th–75th percentile).

^†^ Data are expressed as mean (SD).

**Figure 2:**
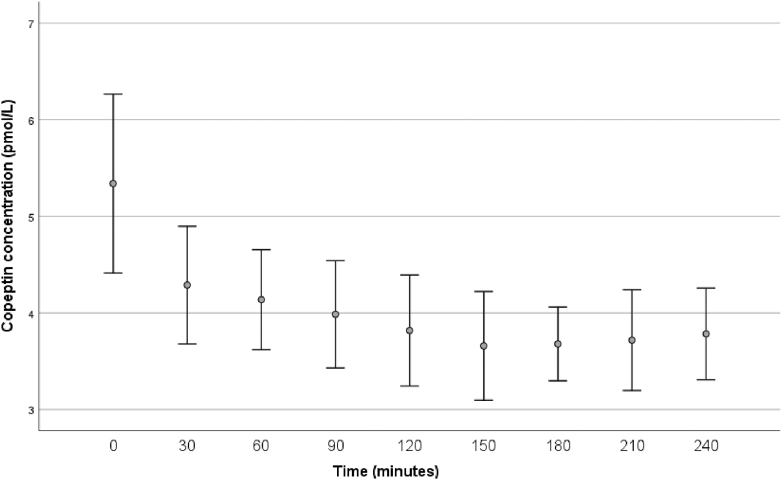
Effects of acute coffee load on plasma copeptin. Plasma copeptin concentration measured at baseline and minutes after 4 dL coffee intake (*n* = 26). Bars illustrating mean and 95% confidence interval. At baseline (‘0’), median plasma copeptin concentration was 4.88 (25th, 75th percentile 3.40–7.26) pmol/L. The lowest plasma copeptin concentration was seen after 150 min with a median copeptin of 3.58 (25th, 75th percentile 2.58–4.36) pmol/L. Copeptin concentration was significantly lower than baseline at all time points (*P* < 0.001 for all).

The mean copeptin concentrations after acute ingestion of 4 dL of coffee are shown in Fig. 2. The copeptin concentration significantly decreased (*P* < 0.001) within 30 min after coffee intake and remained significantly lower than baseline throughout the 4 h measurement period (all *P* < 0.001). The lowest copeptin concentration was observed after 150 min with a median reduction from baseline of 27% (3.58 pmol/L).

**Figure 3:**
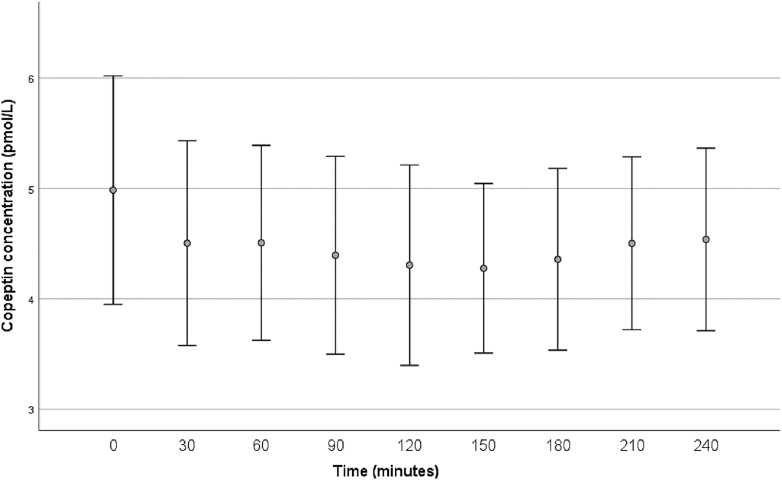
Effects of acute intake of 10 mL water on plasma copeptin (*n* = 26). Bars illustrating mean and 95% confidence interval. At baseline (‘0’), median plasma copeptin concentration was 3.76 (25th, 75th percentile 3.06–7.31) pmol/L. The lowest plasma copeptin concentration was seen after 120 min with a median copeptin of 3.38 (25th, 75th percentile 2.80–5.10) pmol/L. Copeptin concentration was significantly lower than baseline at 120, 150, and 180 min (*P* = 0.006, 0.028, and 0.036, respectively), and nonsignificantly lower at the other time points.

During the control procedure (ingestion of 10 mL of water), the copeptin concentration decreased to a minimum of, in median, 3.38 (2.80, 5.10) after 120 min (Fig. 3). The copeptin change at 120, 150, and 180 min was statistically significant (*P* = 0.006, 0.028, and 0.036, respectively), whereas the change was nonsignificant at other time points.

In a post hoc analysis, the participants were divided into subgroups of higher (≥400 mOsm/kg H_2_O) or lower urine osmolality (<400 mOsm/kg H_2_O). The copeptin concentration was significantly higher at baseline in the group with higher urine osmolality than in the group with lower urine osmolality. In the group with higher urine osmolality (*n* = 12), the median copeptin concentration after coffee intake was significantly lower than baseline at all time points. Meanwhile, in the group with lower urine osmolality (*n* = 14), the median copeptin concentration after coffee intake was significantly lower than baseline at all time points except at 60 min (Supplemental Fig. 1A and B).

## Discussion

Our main finding is that coffee consumption at a population level is negatively associated with copeptin concentration, and that the acute intake of coffee has a copeptin-lowering effect. In the population-based MOS, we found a significant association between increasing coffee intake and decreasing copeptin concentration independently of several confounders known to be associated with coffee intake and copeptin concentration. When the analysis was adjusted for either drinking water intake or total fluid intake, the results were maintained, indicating an association between coffee intake and copeptin independent of the hydration level. However, considering that habitual water intake is a strong determinant of vasopressin and copeptin concentration (18), we chose to stratify our analyses based on fluid intake. This resulted in much smaller effect sizes and the loss of significant associations between coffee intake and copeptin in the upper tertile of drinking water intake and the upper two tertiles of total fluid intake. However, associations were evident in the least hydrated individuals. Thus, we concluded that the association between higher coffee intake and lower copeptin concentration is driven by the least hydrated individuals. This may be seen as contradictory, given that caffeine has diuretic properties, which would, in turn, increase the copeptin concentration. However, the diuretic effect of coffee intake gradually decreases with increasing regular coffee intake (habituation). Although caffeine has diuretic properties, regular coffee consumption has not been associated with dehydration markers (16). Therefore, this mechanism could contribute to a hydrating effect, as well as a copeptin-lowering effect, of chronically increased coffee intake. However, the amount of coffee ingested per day is not necessarily related to how often/regularly coffee is ingested. In this study, we had no data on the frequency of coffee intake (e.g., frequent smaller amounts daily or larger amounts occasionally). It is reasonable to assume that most individuals in tertile 2 and 3 were regular coffee drinkers and that the habituation effect would be equal in both groups. Considering that the association between copeptin concentrations decreased significantly per coffee tertile increase, i.e., also between tertile 2 and 3, the habituation effect alone could not explain our results.

The acute coffee experiment also indicates that coffee intake has a copeptin-lowering effect. After the acute ingestion of 4 dL of coffee, the copeptin concentration decreased immediately and remained significantly lower during the whole measurement period (4 h). The epidemiological finding that the copeptin-lowering effect of coffee is driven by the least hydrated individuals is also supported by the sub-analysis of individuals with lower versus higher urine osmolality in the experimental study. In this analysis, we found a significant copeptin-lowering effect in both groups. However, the effect was more pronounced in the group with higher urine osmolality, representing the less hydrated individuals. This finding suggests that well-hydrated individuals are less susceptible to a possible copeptin-lowering effect of coffee. Although the copeptin-lowering effect of coffee could be partly attributed to specific coffee components, this sub-analysis points at the fact that the water component of coffee is the main reason why coffee is linked to a decrease in copeptin concentration.

Given our experimental study design, we could not elucidate whether copeptin reduction is related to an effect from coffee compounds or a hydration effect driven by the water component of coffee and thus acute volume expansion, which is known to rapidly decrease vasopressin secretion (27). Previously, it has been found that the acute intake of 1 L of water decreases copeptin by, in median, 2.28 pmol/L (i.e., almost twice the reduction observed after the intake of 4 dL of coffee), which could imply a relatively equal effect of acute coffee and water intake on copeptin concentration, thus pointing at the water component of coffee as the main contributor of the copeptin-lowering effect (18). Of note, a slight copeptin-lowering effect from the intake of 10 mL of water was also observed. A volume of 10 mL is not enough to cause volume expansion or change in plasma osmolality. Therefore, we concluded that other mechanisms are involved in this reaction. According to previous studies, plasma vasopressin rapidly decreases following drinking, even before any change in plasma osmolality can be detected (28, 29). Vasopressin neurons have been shown to respond to water intake by rapidly decreasing activity, and this system involves the activation of the lamina terminalis (30). The activation of oropharyngeal and gut receptors has been proposed as a signaling pathway in this, seemingly, reflex mechanism (31). In this study, we can confirm that even a very small volume of water can slightly suppress vasopressin release in healthy adults. However, a greater copeptin suppression was observed after the acute ingestion of 4 dL of coffee (and an even greater decrease was seen after the acute ingestion of 1 L of water (18)). From our data, we concluded that a small component of the copeptin-lowering effect from the oral intake of liquids is most probably a result of a reflex mechanism. However, another factor that could have influenced our results to some extent is the circadian variation of copeptin. The copeptin concentration peaks at night and early morning and nadirs in the late afternoon (32).

The results from our observational data do not allow us to determine whether chronically high coffee intake is associated with low copeptin because of coffee compounds or whether the association is related to the habit of drinking any liquid at all. With the proposed oropharyngeal/gut reflex in mind, one could speculate that individuals with a frequent drinking pattern of any liquid, independent of the volume ingested, would tend to have lower copeptin concentration.

An interplay between the most well-known coffee compound (caffeine) and vasopressin is suggested in the literature. According to previous studies, the effect of caffeine on vasopressin activity is mediated through cyclic adenosine monophosphate (cAMP) (33). Vasopressin exerts its renal effects through V2R, which, in turn, transmit intracellular signals via cAMP. Caffeine is thought to slow down cAMP degradation, which would, in turn, amplify vasopressin’s effect (33). Then, an increased vasopressin effect could be speculated to, in the long term, lower vasopressin/copeptin concentrations through a negative feedback mechanism (13). However, this possible effect would probably not be involved in the acute copeptin decrease seen after coffee intake, but could only explain any long-term effects on copeptin concentration among coffee drinkers.

### Clinical relevance

Considering that coffee is widely consumed, its possible physiological and health effects are of great relevance. Vasopressin and coffee have both been shown to correlate with several important cardiovascular diseases and conditions. The possible links between the vasopressin system and coffee intake need to be studied further. More specifically, the results of this study could be of relevance for conditions that predispose to dehydration and elevated vasopressin secretion (e.g., dysregulated diabetes mellitus), or conditions that require the vasopressin agonist desmopressin (e.g., vasopressin deficiency or nocturia). If there is a causal link between coffee compounds and decreased vasopressin secretion because of mechanisms that slow down vasopressin degradation, one could speculate that desmopressin-treated individuals drinking coffee would need lower doses of vasopressin agonists, and that it would be wise to keep coffee intake at an even level to possibly avoid fluctuations in vasopressin/desmopressin concentrations.

### Strengths and limitations

In this study, observational data were obtained from a high-quality population-based study, making the results robust and reliable. Although the experimental study was small, the random-order cross-over design with a 3-week washout period reduced confounding and many possible biasing effects.

Even if evidence of an association between coffee consumption and copeptin is observed in our observational data, nothing can be concluded with regard to causality in a cross-sectional analysis. There is also a possibility that other unknown confounding factors, apart from the ones tested, may have affected the results. The experimental study is an attempt to approach this problem. Unfortunately, inclusion in this study was prematurely stopped because of gastrointestinal side effects, and this study predominantly included women.

## Conclusion

The present study supports an independent relationship between higher coffee consumption and lower copeptin concentration. Moreover, the acute ingestion of coffee lowers copeptin concentration rapidly and persistently. We postulate that coffee may interact with the vasopressin system either through volume load, coffee-specific pathways, or oropharyngeal/gut reflexes responding to the regular intake of liquids.

## Supplementary materials



## Declaration of interest

Dr Enhörning has participated and participates in ongoing research trials partly funded by Danone Research. Dr Melander has received a research grant and consultancy fee from Danone Research. The authors report no other competing interests in this work.

## Funding

This work was supported by the Swedish Research Council (2018-02784; 2018-02837; 2021-03291), the Swedish Heart and Lung Foundation (20200711), the regional Region Skane County Council ALF grant (2018-0148; 2022-0258), the Novo Nordisk Foundation (NNF20OC0063886), and the Swedish Diabetes Foundation (DIA 2018-375). MOS has additionally been funded by the Swedish Research Council (2013-2756), the Swedish Heart and Lung Foundation (2015-0427), and by funds from the local Region Skane County Council (ALF) to Peter M Nilsson and Fredrika Schill (‘ST-ALF’). We also acknowledge the financial support for collection and management of dietary data in MOS to Marju Orho-Melander from the European Research Council (ERC-CoG-2014-649021).
