# Supplementary material for: Coffee intake and the vasopressin system: an epidemiological and experimental study
Source: Endocr Connect. 2025 Sep 5;14(9):e250100. doi: 10.1530/EC-25-0100 (PMC12421983; doi:10.1530/EC-25-0100)
Supplement: Supplementary file 1 [file supplementary_materials.pdf]

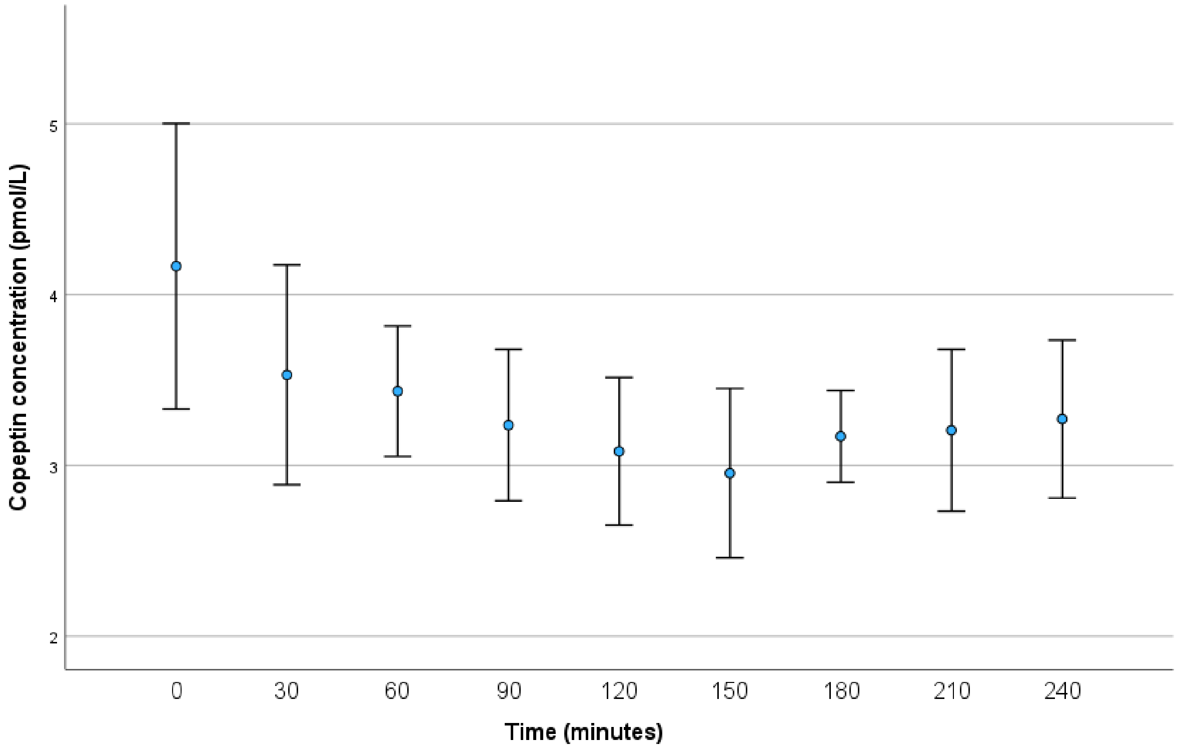

Supplemental figure 1A. Effects of acute coffee load on plasma copeptin among participants with lower urine osmolality (< 400 mosm/kg H2O, n=14). Plasma copeptin concentration measured at baseline and minutes after 4 dL coffee intake. Bars illustrating mean and 95% confidence interval. At baseline ("0") median plasma copeptin concentration was 3.86 (25th, 75th percentile 3.19-4.87) pmol/L. The lowest plasma copeptin concentration was seen after 150 minutes with a median copeptin of 2.62 (25th, 75th percentile 2.45-3.78) pmol/L. Median copeptin concentration was significantly lower than baseline at all time points ($P<0.05$), except for at 60 minutes ($p = 0.056$).

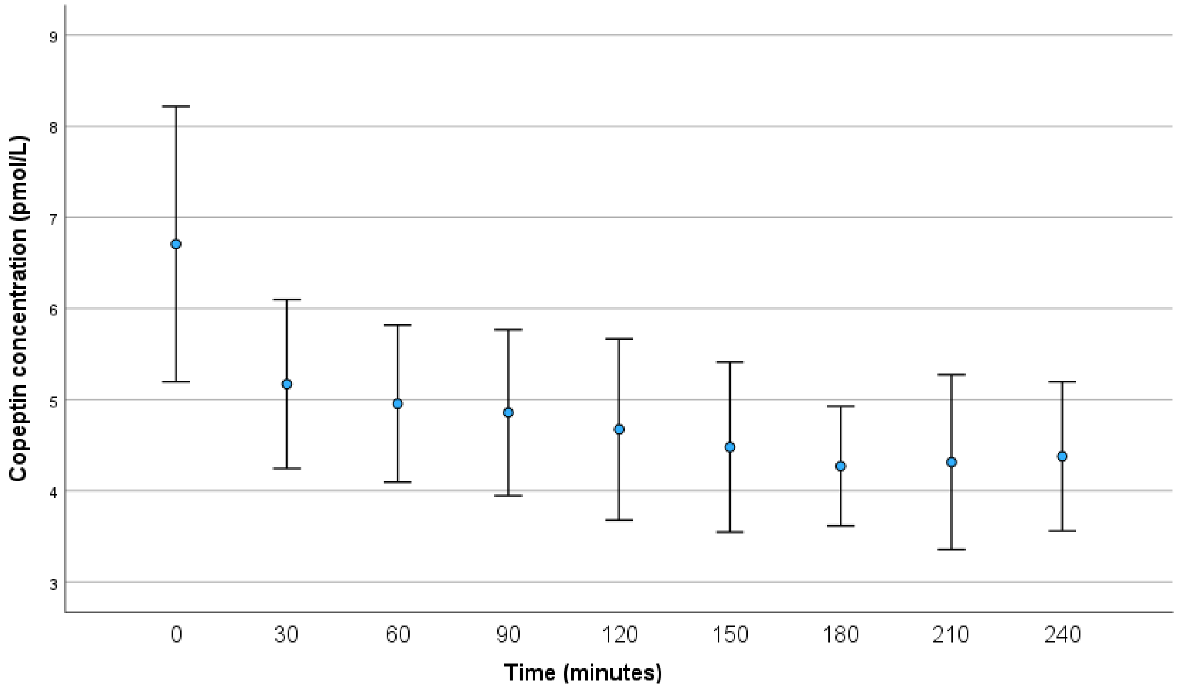

Supplemental figure 1B. Effects of acute coffee load on plasma copeptin among participants with higher urine osmolality (>/= 400 mosm/kg H2O, n=12). Plasma copeptin concentration measured at baseline and minutes after 4 dL coffee intake. Bars illustrating mean and 95% confidence interval. At baseline ("0") median plasma copeptin concentration was 6.52 (25th, 75th percentile 5.52-8.71) pmol/L. The lowest plasma copeptin concentration was seen after 210 minutes with a median copeptin of 4.09 (25th, 75th percentile 3.10-4.84) pmol/L. Median copeptin concentration was significantly lower than baseline at all time points ($P<0.05$ for all).

Supplemental table 1. Baseline characteristics of included and excluded participants.

	**Included participants** (N= 3270)	**Excluded participants** (N= 2018)	**All participants** (N= 5288)
**Coffee intake,** g/d[1]	281.9 (25.0, 475.0)	275.0 (0.0, 475.0)	275.0 (15.0, 475.0)
N	3270	551	3821
**Males**	300.0 (50.0, 500.0)	275.0 (0.0, 500.0)	300.0 (50.0, 500.0)
**Females**	275.0 (0.0, 450.0)	262.0 (0.0, 450.0)	275.0 (0.0, 450.0)
**Copeptin,** pmol/L[1]	5.8 (3.8, 9.0)	6.3 (4.2, 9.7)	5.9 (3.9, 9.2)
N	3270	1327	4597
**Copeptin men,** pmol/L[1]	7.3 (5.0, 10.8)	8.0 (5.4, 11.2)	7.5 (5.2, 11.0)
**Copeptin women,** pmol/L[1]	4.6 (3.3, 6.9)	5.0 (3.5, 7.7)	4.7 (3.3, 7.2)
**Drinking water intake,** g/day[1]	575.0 (250.0, 1000.0)	540.0 (207.5, 962.5)	575.0 (250.0, 1000.0)
N	3270	551	3821
**Total fluid intake**, g/day	2227.6 (821.3)	2163.3 (824.4)	2218.3 (822.0)
N	3270	551	3821
**Age**, years	43.2 (14.6)	40.2 (14.7)	42.1 (14.7)
N	3270	1999	5269
**Current smoking,** %	12.9	22.5	19.4
N	3133	1683	4816
**Men,** %	46.7	51.0	48.3
N	3270	2007	5277
**Glucose,** mmol/L	5.4 (1.0)	5.4 (1.2)	5.4 (1.1)
N	3270	1981	5251
**Creatinine,** µmol/L	77.6 (17.4)	77.2 (15.6)	77.5 (16.7)
N	3270	1964	5234
**BMI,** kg/m^2	26.0 (4.6)	26.5 (5.1)	26.2 (4.8)
N	3270	1993	5263
**HDL,** mmol/L	1.6 (0.5)	1.6 (0.5)	1.6 (0.5)
N	3270	1966	5236
**LDL,** mmol/L	3.2 (1.0)	3.1 (1.0)	3.1 (1.0)
N	3270	1964	5234
**Triglycerides,** mmol/L	1.1 (0.7)	1.2 (1.2)	1.2 (1.0)
N	3270	1949	5219
**Systolic blood pressure,** mmHg	119.2 (15.9)	119.3 (16.3)	119.3 (16.1)
N	3270	1793	5063
**Diastolic blood pressure,** mmHg[1]	74.4 (1.0)	74.4 (10.5)	74.4 (10.2)
N	3270	1793	5063

Data expressed as mean (standard deviation) if nothing else specified.

[1] Median (25th, 75th percentile)
